# Milk Fat Globule-EGF Factor 8 Contributes to Progression of Hepatocellular Carcinoma

**DOI:** 10.3390/cancers12020403

**Published:** 2020-02-10

**Authors:** Duck Sung Ko, Su Hyun Kim, Ji Young Park, Gyunggyu Lee, Hyo Jin Kim, Gyeongmin Kim, Kyun You Chi, Ilsoo Kim, Jinseok Lee, Kyu-Yeoun Won, Jiyou Han, Jeongsang Son, Dong-Hun Woo, Choongseong Han, Jong-Hoon Kim

**Affiliations:** 1 Laboratory of Stem Cells and Tissue Regeneration, Department of Biotechnology, College of Life Sciences and Biotechnology, Korea University, Seoul 02841, Korea; koducksung@gmail.com (D.S.K.); prssjy@hanmail.net (J.Y.P.); chloe.gyu@gmail.com (G.L.); 010hyojin@naver.com (H.J.K.); kkkjjj0815@naver.com (G.K.); viclavoc@naver.com (K.Y.C.); kis8295@gmail.com (I.K.); wlstjr0806@naver.com (J.L.); sllzzz@naver.com (J.S.); 2 Department of Pathology, Kyung Hee University Hospital at Gangdong, College of Medicine, Kyung Hee University, Seoul 05278, Korea; wonlover2@hanmail.net (S.H.K.); wonki96@hanmail.net (K.-Y.W.); 3 Department of Biological Sciences, Hyupsung University, Hwasung-si 18330, Korea; hanjiyou12@hanmail.net; 4 Department of New Drug Development, NEXEL Co., Ltd., Seoul 07802, Korea; dhwoo@nexel.co.kr (D.-H.W.); nexelceo@nexel.co.kr (C.H.)

**Keywords:** Hepatocellular carcinoma, milk fat globule-EGF factor 8, proliferation, migration

## Abstract

Milk fat globule-EGF factor 8 (MFG-E8) is an anti-inflammatory glycoprotein that mediates a wide spectrum of pathophysiological processes. MFG-E8 has been studied as a key regulator of cancer cell invasion, migration, and proliferation in different tissues and organs. However, potential roles of MFG-E8 in the growth and progression of liver cancer have not been investigated to date. Here, we analyzed 33 human hepatocellular carcinoma (HCC) samples and found that levels of MFG-E8 expression were significantly higher in HCC cells than in normal liver tissues. In addition, our in vitro gain-of-function study in three different HCC cell lines revealed that overexpression of MFG-E8 promoted the proliferation and migration of HCC cells, as determined by RT-qPCR, MTT assays, and wound healing analyses. Conversely, an MFG-E8 loss-of function study showed that proliferation capacity was significantly reduced by MFG-E8 knockdown in HCC cells. Additionally, MFG-E8 activity-neutralizing antibodies profoundly inhibited both migration and proliferation of HCC cells, attenuating their tumorigenic properties. These reductions in migration and proliferation were rescued by treatment of HCC cells with recombinant MFG-E8 protein. Furthermore, an in vivo HCC xenograft study showed that the number of proliferating HCC cells and tumor volume/weight were all significantly increased by MFG-E8 overexpression, compared to control mice. These results clearly show that MFG-E8 plays an important role in HCC progression and may provide a basis for future mechanistic studies and new strategies for the treatment of liver cancer.

## 1. Introduction

Hepatocellular carcinoma (HCC) is the fifth most common cancer worldwide and the third-most common cause of cancer mortality [1]. Although multidisciplinary therapeutic strategies have been proven effective for HCC treatments, the five-year survival of HCC patients remains poor due to frequent tumor recurrences after surgery [2]. Therefore, elucidating the molecular mechanism underlying HCC tumorigenesis and identifying novel therapeutic targets are both of great significance in improving the overall prognosis of patients with HCC.

Milk fat globule-EGF factor 8 (MFG-E8) is a glycoprotein initially identified as a component of milk fat globules secreted from mammary epithelial cells [3]. Human MFG-E8 consists of an N-terminal EGF-like domain which contains an integrin-binding RGD motif, and two repeated C-terminal discoidin/F5/8C domains that bind phosphatidylserine [3]. Because phosphatidylserine is exposed on the surfaces of apoptotic cells, MFG-E8 has been studied as an opsonin that mediates clearance of dying cells via integrin-expressing phagocytes [4,5]. MFG-E8 is also expressed in many cell types, including mammary epithelial and myoepithelial cells, dendritic cells, endothelial cells, intestinal cells, and retinal epithelial cells. It has been demonstrated that MFG-E8 regulates cellular proliferation, differentiation, and migration under various pathophysiological conditions, including sepsis and age-related diseases [6,7]. We also showed that MFG-E8 is secreted from human umbilical cord mesenchymal stem cells and strongly inhibits the activation of hepatic stellate cells, reducing liver fibrosis both in vitro and in vivo [8].

Recent studies have revealed that MFG-E8 is involved in the progression of a variety of tumors, including breast cancer, ovarian cancer, and colorectal cancer [9,10,11]. MFG-E8 expression is significantly upregulated in colorectal cancer, oral squamous cell carcinoma, and melanoma, compared with normal tissues [11,12,13]. A previous study found that there was a significantly higher rate of high MFG-E8 expression in the esophageal tumor tissues of patients treated with neoadjuvant chemotherapy (NAC), compared to those not treated with NAC [14]. They also observed the increased expression of MFG-E8 in esophageal cancer cell lines after treatment with chemotherapeutic drugs. Another study using MFG-E8 knockout mice showed that MFG-E8 conferred anticancer drug resistance to cancer stem cells by activating Stat3 and Hedgehog signaling pathways [15], suggesting the potential role of MFG-E8 in tumor resistance to chemotherapy. In addition, knockdown of the MFG-E8 gene reduces the tumorigenic properties of human melanoma cells [13]. Induction of systemic MFG-E8 blockade by the infusion of anti-MFG-E8 antibodies significantly intensifies the antitumor activities of various therapeutic regimens in mouse xenografts of colon carcinoma, melanoma, thymoma, and fibrosarcoma [16]. However, the role of MFG-E8 in the development and progression of liver cancer has yet to be elucidated. 

In this study, we investigated the role of MFG-E8 in HCC using human HCC samples, three different HCC cell lines, and a xenograft animal model of HCC. Our data provide the first direct evidence that MFG-E8 is highly expressed in human HCC tissues and positively regulates the progression of HCC both in vitro and in vivo.

## 2. Results

### 2.1. MFG-E8 Expression is Significantly Upregulated in Liver Tumor Tissues and HCC Cell Lines

**Figure 1:**
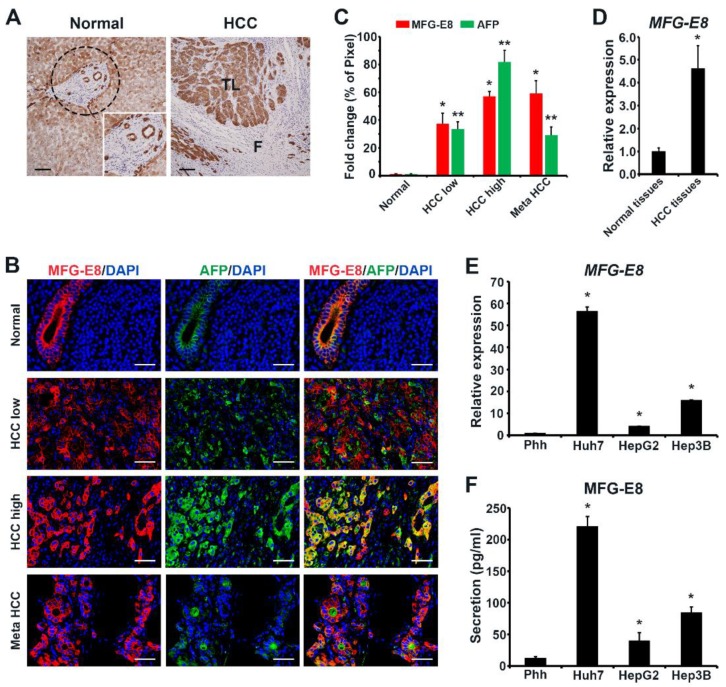
Expression of milk fat globule-EGF factor 8 (MFG-E8)in clinical hepatocellular carcinoma (HCC) tissues and HCC cell lines. (**A**) Immunohistochemical staining of MFG-E8 in HCC tissues and normal liver tissues obtained from patients with HCC. Inset, enlarged image of the dashed circular area. TL, tumor lobule; F, fibrous septa. Scale bars, 100 μm. (**B**) Representative immunofluorescence images of primary and metastatic HCC tissues, showing the expression of MFG-E8 and AFP. Cell nuclei were stained with DAPI. Scale bars, 50 μm. Quantitation of immunoreactive cells are shown in (**C**). The percentages of positive immunofluorescence signal intensities of total image (% of pixel) were measured using ImageJ software and expressed as relative values to those in normal tissues. (**D**,**E**) The mRNA expression levels of MFG-E8 in normal tissues and HCC tissues (**D**) and in human primary hepatocytes (Phh) and HCC cell lines (Huh7, HepG2, and Hep3B) (**E**). mRNA expression data were normalized to GAPDH levels and expressed as relative values. (**F**) The levels of MFG-E8 protein secreted from human Phh and HCC cells. Data represent the mean ± S.D. * *p* < 0.05 versus normal tissue (**C**,**D**) and Phh (**E**,**F**) by a two-tailed Student’s *t*-test.

The parenchyma of normal liver tissues showed diffuse and moderate positive cytoplasmic immunostaining for MFG-E8 (Figure 1A, left). The area of the stroma in portal tract was negative for MFG-E8 expression, but the bile ducts showed strong positive signals for MFG-E8 (Figure 1A, dashed circle in left panel and inset). Notably, a profound increase in MFG-E8 was detected in HCC tissues (Figure 1A, right). Strong MFG-E8 signals were primarily confined to tumor lobules surrounded by thick fibrous septa that were negative for MFG-E8. Based on double immunofluorescence staining, we observed that normal tissues showed strong positive signals of MFG-E8 in ductal areas (Figure 1B: Normal), as observed in Figure 1A. The expression levels of MFG-E8 and α-fetoprotein (AFP), a marker of HCC, were both significantly increased in HCC tissues (Figure 1B,C) and the immunofluorescence signals were overlapped in areas of high malignancy (Figure 1B: HCC high). Prominent MEG-E8 signals were also seen in metastatic HCC tissues, but low signals of AFP were detected in these areas (Figure 1B: Meta HCC). RT-qPCR analysis also revealed that the expression levels of MFG-E8 in human HCC tissues were significantly upregulated compared to levels in normal liver tissues (Figure 1D). We also investigated the expression of MFG-E8 in three different HCC cell lines (Huh7, HepG2, and Hep3B). Consistent with the tissue data, RT-qPCR and ELISA results demonstrated that the levels of gene expression (Figure 1E) and protein secretion (Figure 1F) of MFG-E8 were both significantly increased in HCC cell lines compared to primary normal hepatocytes, although the levels differed between the cell lines. In particular, Huh7 cells showed the highest level of MFG-E8 expression and secretion among the three HCC cell lines tested.

### 2.2. MFG-E8 Promotes Proliferation of HCC Cells

**Figure 2:**
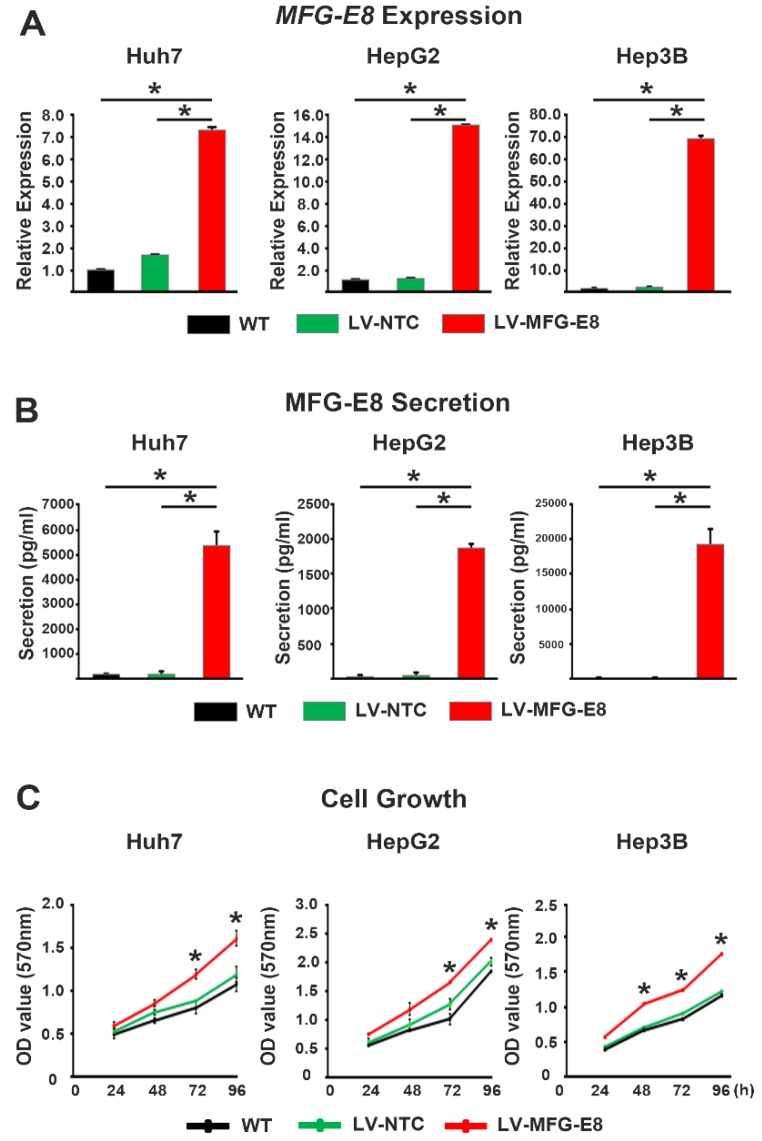
Effects of MFG-E8 overexpression on HCC cells proliferation. (**A**,**B**) The levels of mRNA (**A**) and secreted protein (**B**) of MFG-E8 from HCC cells after transfection with nontargeting control lentivirus (LV-NTC) or MFG-E8-overexpressing lentivirus (LV-MFG-E8). WT, untransfected cells. (**C**) Growth curves of HCC cells after transfection with LV-NTC or LV-MFG-E8. * *p* < 0.05, a two-tailed Student’s *t*-test.

We next investigated the effects of MFG-E8 on the proliferation of HCC cells in vitro in three different hepatic tumor cell lines (Huh7, HepG2, and Hep3B) using both gain- and loss-of-function approaches. The gain-of-function study was performed by overexpressing MFG-E8 in three HCC cell lines using a lentiviral vector (LV-MFG-E8). RT-qPCR and ELISA results confirmed that the levels of MFG-E8 expression and secretion significantly and profoundly increased after MFG-E8 overexpression in all three cell lines compared to both the control cell line transfected with a nontargeting control lentivirus vector (LV-NTC) and untransfected wild-type (WT) cells (Figure 2A,B). Importantly, a cell proliferation assay revealed that the MFG-E8 gain of function (LV-MFG-E8) led to significant increases in tumor cell proliferation in all cell lines tested compared to control (LV-NTC) and WT cells (Figure 2C).

**Figure 3:**
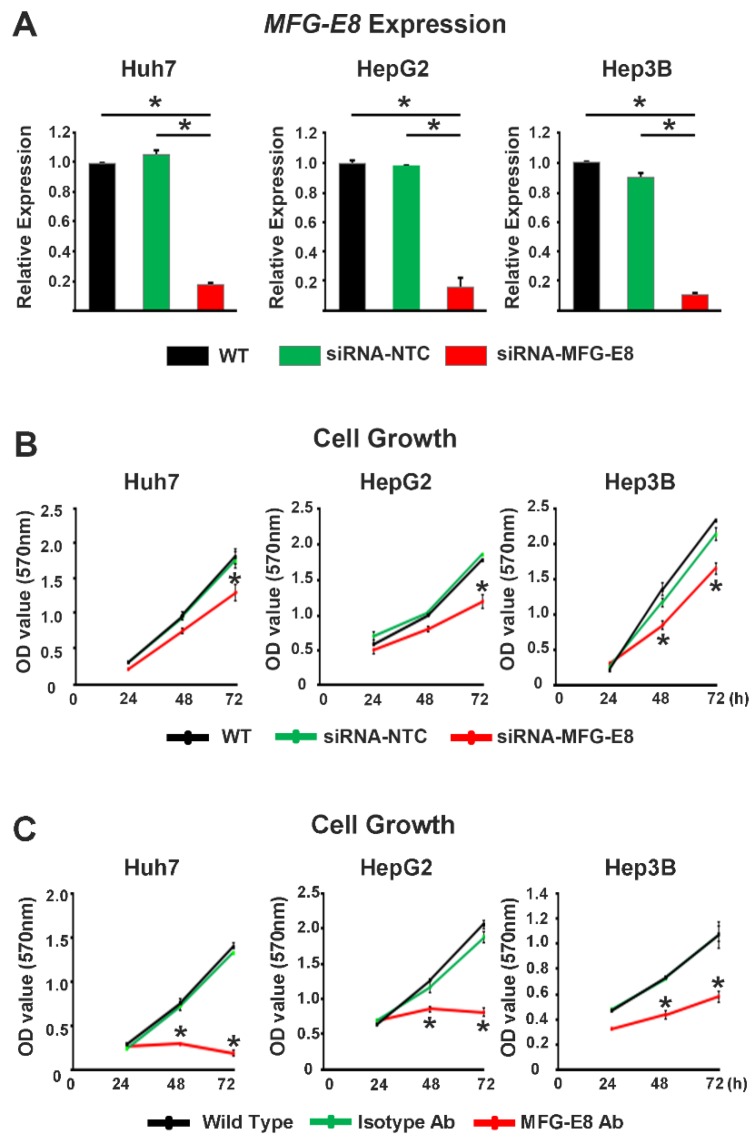
Effects of MFG-E8 knockdown and activity inhibition on HCC cell proliferation. (**A**) The mRNA expression levels of MFG-E8 in HCC cells after transfection with nontargeting control siRNA lentivirus (siRNA-NTC) or MFG-E8-siRNA lentivirus (siRNA-MFG-E8). (**B**) The proliferation of HCC cells was evaluated at different time points in cells transfected with siRNA-NTC or siRNA-MFG-E8. (**C**) Growth curves of HCC cells cultured in the absence (untreated) and presence of isotype control IgG or anti-MFG-E8 antibody (MFG-E8 Ab). * *p* < 0.05 versus WT, a two-tailed Student’s *t*-test.

The loss-of-function study was conducted using a siRNA that silences the expression of MFG-E8 and an anti-MFG-E8 antibody that directly inhibits MFG-E8 activity. In all three HCC cell lines, knockdown of MFG-E8 by siRNA interference (siRNA-MFG-E8) significantly and markedly reduced the expression levels of MFG-E8 compared to control (siRNA-NTC) and WT cells (Figure 3A). The silencing of MFG-E8 expression in HCC cell lines resulted in a significant attenuation of cell growth 3 days post-infection compared with control (WT and siRNA-NTC) treatments (Figure 3B). Interestingly, direct inhibition of MFG-E8 activity using anti-MFG-E8 antibodies showed a more potent antiproliferative effect in HCC cell lines, almost completely abolishing HCC cell growth 24–72 h after antibody treatment (Figure 3C).

### 2.3. MFG-E8 Promotes Migration of HCC Cells

**Figure 4:**
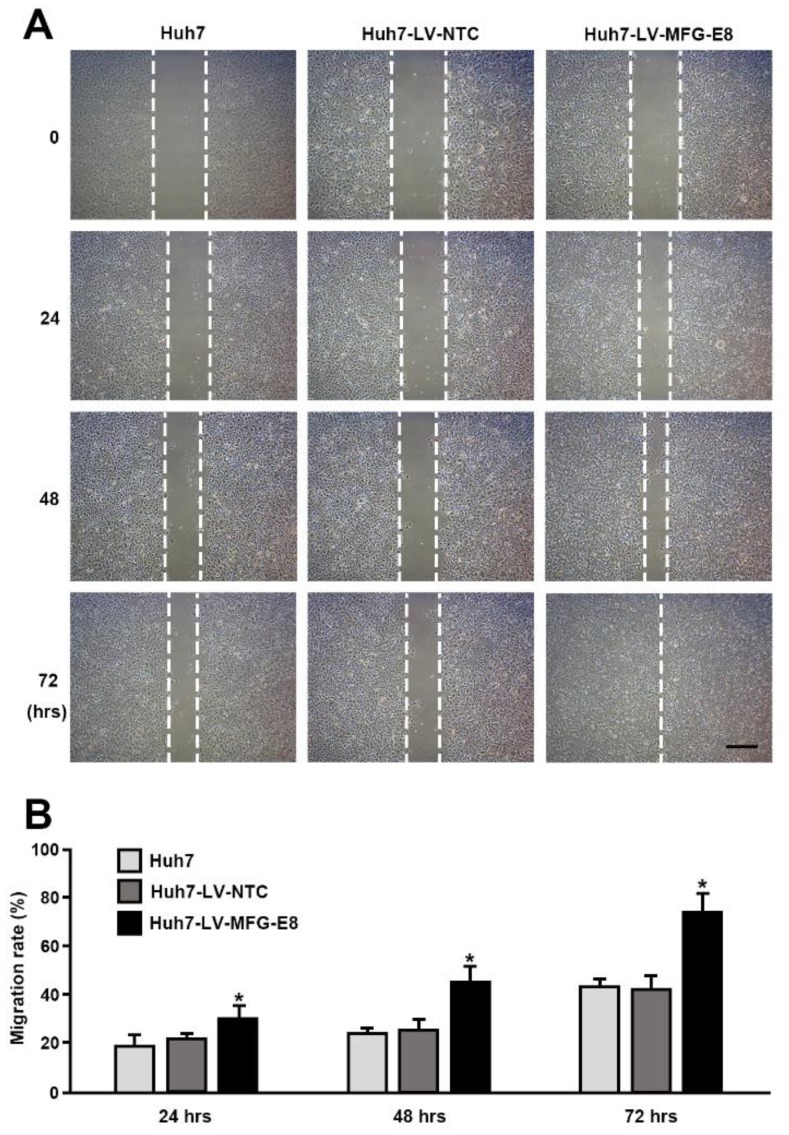
Effects of MFG-E8 overexpression on HCC cells migration. (**A**) Migration of Huh7 cells transfected with either nontargeting control lentivirus vector (LV-NTC) or LV-MFG-E8 was evaluated by a scratch assay; phase-contrast images were taken 24, 48, 72, and 96 h after making the mechanical scratch wound in vitro. The migration rates are shown in (**B**). Data represent the mean ± S.D. * *p* < 0.05, a two-tailed Student’s *t*-test. Scale bars, 200 μm.

Cell migration is involved in many pathological processes, including invasion, angiogenesis, and metastasis, which are essential activities of tumor cells. Wound healing assays are widely used methods for investigating of carcinoma cell migration [12]. We examined the effects of MFG-E8 on the migration of HCC cells in response to a mechanical scratch wound in vitro. The results showed that MFG-E8 overexpression (LV-MFG-E8) significantly increased the migratory ability of Huh7 cells, closing the open scratched area faster than control LV-NTC and WT cells (Figure 4A,B; data obtained from HepG2 and Hep3B are provided in Figure S1).

**Figure 5:**
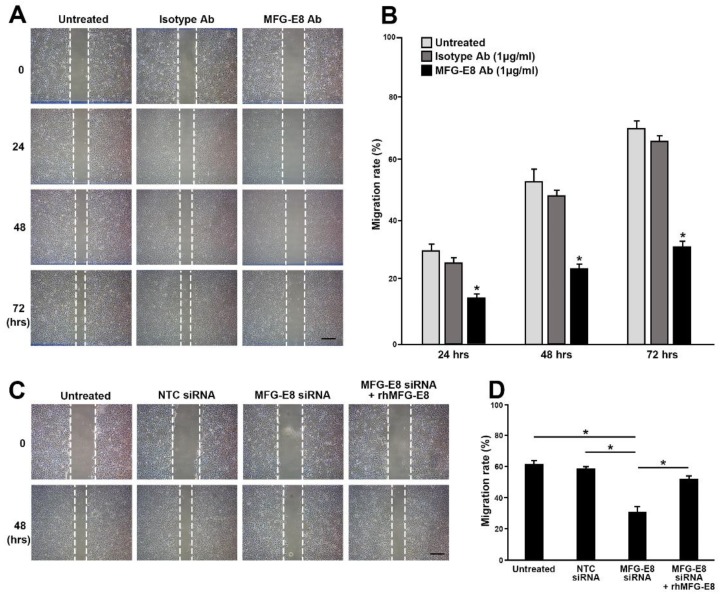
HCC cells migration after inhibiting MFG-E8 activity and silencing the expression of MFG-E8. (**A**) Migration of Huh7 cells treated with either isotype IgG or anti-MFG-E8 antibody was evaluated by a scratch assay; phase-contrast images were taken 24, 48, 72, and 96 h after making the mechanical scratch wound in vitro. The migration rates are shown in (**B**). (**C**,**D**) Migration of Huh7 after transfection with siRNA-MFG-E8 was analyzed in the absence or present of recombinant human MFG-E8 proteins (rhMFG-E8). The comparison of the migration rate is shown in (**D**). Data represent the mean ± S.D. * *p* < 0.05, a two-tailed Student’s *t*-test. Scale bars, 200 μm.

In contrast, the motility of Huh7 cells was steadily and markedly impaired by treatment with anti-MFG-E8 antibodies (1 μg/mL) during the 72 h of measurement (Figure 5A,B; data obtained from HepG2 and Hep3B are provided in Figures S2 and S3). Silencing of MFG-E8 expression using siRNA (siRNA-MFG-E8) also significantly decreased the migratory capability of Huh7 cells compared with both control siRNA-NTC and WT cells 48 h after transfection (Figure 5C,D; data obtained from HepG2 and Hep3B are provided in Figure S4). However, the reduction in motility of MFG-E8 siRNA-transfected HCC cells was rescued by the addition of recombinant human MFG-E8 protein (rhMFG-E8, 500 ng/mL) (Figure 5C,D).

### 2.4. MFG-E8 Activities Are Involved in the Integrin-Akt Signaling Pathway in HCC Cells

**Figure 6:**
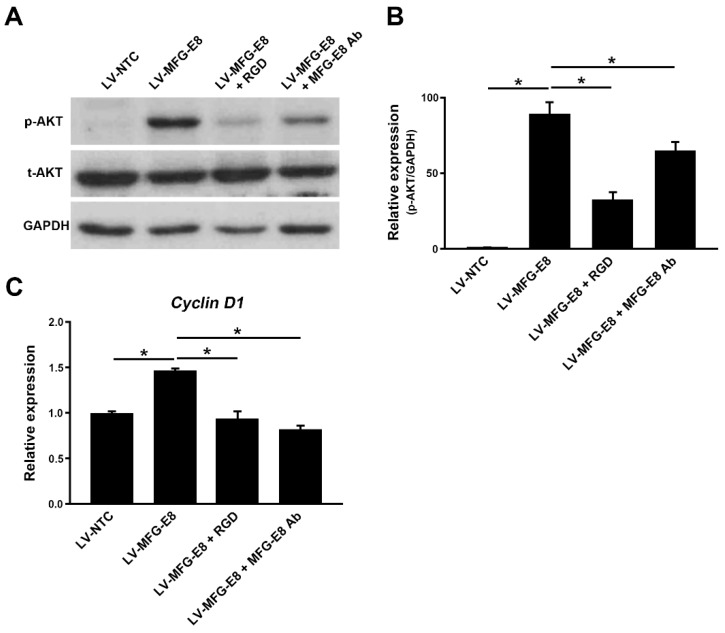
MFG-E8 regulates Akt phosphorylation and Cyclin D1 expression. (**A**) Representative western blots for the phosphorylation of Akt in control (LV-NTC) and MFG-E8 overexpressing (LV-MFG-E8) HCC cells (Huh 7). LV-MFG-E8-HCC cells were cultured in presence or absence of MFG-E8 antibodies (MFG-E8 Ab, 1 μg/mL) or GRGDSP peptide (RGD, 100 μM) for 24 h. (**B**) Quantitation of the Akt phosphorylation in HCC cells cultured under different conditions. (**C**) The RNA expression levels of cyclin D1 (*CCND1*) in HCC cells. Cells were cultured and treated as in (**A**). * *p* < 0.05, a two-tailed Student’s *t*-test.

We next investigated the potential signaling pathways mediated by MFG-E8 in HCC cells. Western blot analyses showed that phosphorylation of Akt was markedly increased in the HCC cells overexpressing MFG-E8 (LV-MFG-E8), compared with the control cells (LV-NTC) (Figure 6A,B). However, the enhanced phosphorylation of Akt was decreased by inhibiting MFG-E8 activity using anti-MFG-E8 antibodies in MFG-E8 overexpressing HCC cells (Figure 6A,B). Interference with MFG-E8 binding to integrins using a synthetic integrin-blocking RGD-based peptide (GRGDSP) also reduced the phosphorylation of Akt (Figure 6A,B), suggesting that the Akt phosphorylation was mediated by the interaction between the RGD modif of MFG-E8 and integrins. In addition, the expression of cyclin D1 (CCND1) was upregulated in HCC cells overexpressing MFG-E8, compared with the control cells (NTC) (Figure 6C). However, the upregulation of CCND1 was inhibited by the treatment of HCC cells with MFG-E8 antibodies or RGD-peptides. These data suggest that the action of MFG-E8 on HCC cells is mediated through the integrin-Akt signaling pathway.

### 2.5. MFG-E8 Promotes HCC Tumor Formation In Vivo

**Figure 7:**
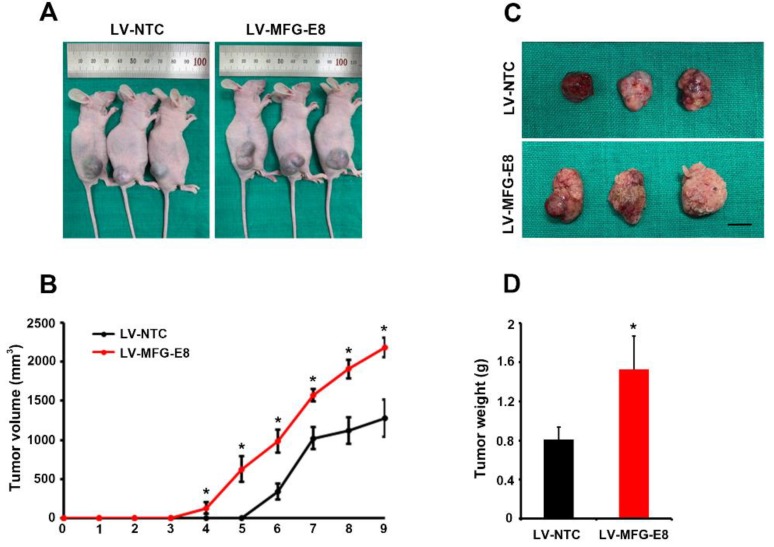
MFG-E8 overexpression promotes growth of HCC xenografts in vivo. (**A**) In vivo visualization of solid tumors in xenograft mice that received Huh7 cells transfected with LV-NTC or LV-MFG-E8. Images were taken 9 weeks after grafting. (**B**) Tumor volume was measured using a slide caliper every week for 9 weeks (n = 3 per group). (**C**) Dissected xenografts generated from Huh7 cells transduced either with LV-NTC or LV-MFG-E8. Scale bars, 10 mm. (**D**) Quantitative analysis of tumor weights 9 weeks after grafting.

**Figure 8:**
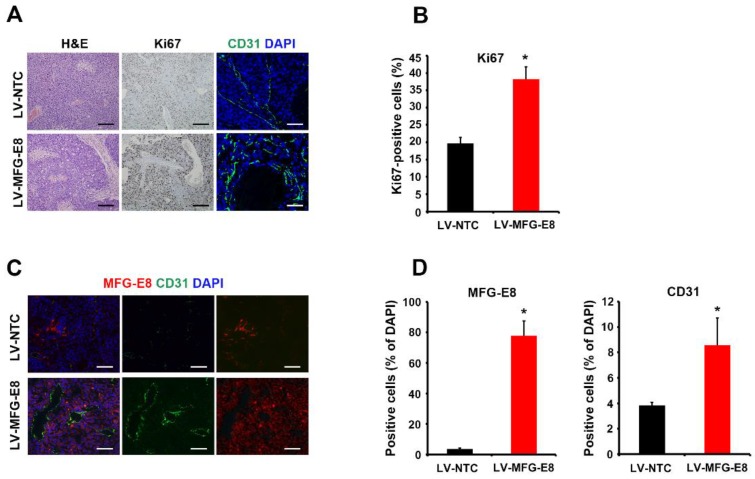
Histological analysis of HCC xenografts. (**A**) Tissue sections were stained with hematoxylin and eosin (H&E), anti-Ki67 antibody, and anti-CD31 antibody. Scale bars, 50 μm. (**B**) Quantitation of Ki67-positive cells. (**C**) Immunofluorescence staining of HCC xenografts for MFG-E8 (red) and CD31 (green). Cells were counterstained with DAPI (blue). Quantitation of immunoreactive cells is shown in (**D**). Scale bars, 50 μm. * *p* < 0.05, two-tailed Student’s *t*-test.

The data obtained from clinical samples and in vitro assays prompted us to investigate whether MFG-E8 promotes tumor formation in a mouse xenograft model. BALB/c nude mice were injected with Huh7 cells transduced with either control lentivirus (LV-NTC) or MFG-E8-overexpressing lentivirus (LV-MFG-E8) into the right flank (n = 3 for each group). Tumor size was measured up to 9 weeks after grafting (Figure 7A,B). As expected, MFG-E8 overexpression resulted in a marked increase in both tumor volume and weight, compared to the control group (Figure 7B–D). Histological analyses of HCC xenografts revealed that the number of Ki-67-positive proliferating cells was significantly greater in MFG-E8-overexpressing HCC xenografts compared with control HCC xenografts (Figure 8A,B). We also observed that cells positive for CD31, a blood endothelial cell marker, were significantly increased in HCC xenografts overexpressing MFG-E8 over control HCC xenografts (Figure 8C,D). The growth of tumor in xenograft animals was also analyzed after injections of two additional HCC cell lines, Hep3B and HepG2, that overexpressed MFG-E8. A similar pattern of increased tumor growth by the MFG-E8 overexpression was observed in the additional xenograft studies (Data obtained from Hep3B and HepG2 are provided in Figure S5).

## 3. Discussion

While previous data revealed a significant correlation of MFG-E8 with the progression of multiple tumor types [9], no available data have been published regarding the tumorigenic activity of MFG-E8 in HCC. High levels of MFG-E8 expression have been correlated to tumor progression via multiple pathways in various cancer types in different tissues, including the salivary gland, thyroid, pancreas, ovary, brain, skin, bladder, and blood [17]. A previous study demonstrated that gene expression of MFG-E8 was significantly increased in tumor tissues from patients with cholangiocarcinoma and suggested that MFG-E8 is a promising biomarker for the management of cholangiocarcinoma [18]. A recent study showed that serum MFG-E8 levels were significantly lower in patients with HCC than in healthy controls, suggesting that serum MFG-E8 could be a feasible biomarker for HCC [19]. In the present study, we compared HCC tissues and adjacent normal tissues from the same patients and showed that MFG-E8 expression was significantly increased in primary and metastatic HCC tissues compared to normal liver tissues. We also observed that the expression of MFG-E8 was upregulated in all three HCC cell lines tested compared to primary hepatocytes. These results are in good agreement with the above-mentioned studies demonstrating a positive correlation of MFG-E8 expression in tissues with tumor progression in other organs [9]. Patients with melanoma who exhibited a high level of MFG-E8 expression had significantly shorter survival periods than those without MFG-E8 expression [17]. Therefore, these findings altogether strongly suggest that MFG-E8 expression may serve as a promising tissue biomarker for both the diagnosis and prognosis of HCC. As described above, a previous study demonstrated that serum levels of MFG-E8 were lower in HCC patients compared with healthy control [19]. However, this study obtained contradictory results from liver tissues, showing that the expression levels of MFG-E8 were higher in HCC tissues than those in normal tissues [19], as we observed in the present study. Our previous study also demonstrated similar conflicting results showing that serum MFG-E8 levels were comparable in normal and cirrhotic patients, while tissue expression of MFG-E8 was reduced profoundly in the liver tissues of patients with cirrhosis [8]. The discrepancy in MFG-E8 levels between liver tissues and serum was possibly due to the production of MFG-E8 from various tissues other than the liver [3]. Currently, there is limited information regarding the expression kinetics of MFG-E8 during tumor progression, not only for HCC but also for other cancer types. Therefore, it will be interesting to investigate the kinetics of MFG-E8 expression in tissues and its levels in serum during the different stages of acute and chronic liver diseases and tumorigenesis.

Our in vitro loss- and gain-of-function analyses clearly showed that MFG-E8 is actively involved in HCC tumor progression. We also showed that MFG-E8 has tumor-promoting effects in HCC using a mouse xenograft model in vivo. In particular, the in vitro proliferation and migration of HCC cells were more profoundly reduced by the direct targeting of MFG-E8 activity using antibodies, compared to knockdown of MFG-E8 expression using siRNA. Previous studies showed that the administration of MFG-E8-specific monoclonal antibodies effectively blocked the tumor-promoting effects of MFG-E8 in ovarian and breast carcinomas [20]. In addition, the systemic blocking of MFG-E8 activities using MFG-E8 antibodies enhanced the therapeutic effects of anticancer regimens in colon carcinoma, melanoma, thymoma, and fibrosarcoma [16]. Thus, our data suggest that anti-MFG-E8 antibodies could serve as a potential therapeutic regimen against HCC progression and metastasis.

The exact mechanism by which MFG-E8 affects tumor progression is not fully understood and remains to be investigated. MFG-E8 plays diverse cellular roles by binding to integrins via its RGD motif. Our previous study also showed that the binding of MFG-E8 to integrin αvβ3 suppressed TGFβ-induced activation of hepatic stellate cells [8]. Integrin αvβ3 is highly expressed in primary liver cancer and is involved in HCC progression [21,22]. In the present study, our data suggest that MFG-E8 induced the integrin-mediated phosphorylation of Akt and upregulated the expression of cyclin D1 in HCC cells. Therefore, MFG-E8 may bind to integrins and activate Akt/Twist signaling pathways, promoting HCC cell growth and migration, as previously suggested in patients with melanoma [13]. Previous studies demonstrated that MFG-E8 promotes vascular endothelial growth factor-dependent neovascularization and angiogenesis, possibly through αvβ3/5 integrin-mediated AKT phosphorylation [23,24]. In the present study, we found that the number of CD31-postive endothelial cells significantly increased in HCC tumors overexpressing MFG-E8 compared with control HCC tumor tissues in mouse xenografts. Thus, it is possible that MFG-E8 contributes to blood vessel formation in HCC tissues, which is a key process in tumor progression. Other potential mechanisms may be involved in the exosome-mediated tumor progression. Exosomes play important roles in the exchange and delivery of mRNA, miRNA, and proteins between cells, thus modulating the microenvironment [25,26]. Tumor-derived exosomes promote the epithelial–mesenchymal transition, tumor angiogenesis, and metastasis [20,27,28]. MFG-E8 is secreted from cells in association with exosomes and may play an important role in exosome secretion and delivery [25,29,30]. Thus, it is possible that MFG-E8 may regulate the HCC microenvironment by mediating exosome trafficking. Further studies regarding cellular and molecular events are needed to elucidate the exact role of MFG-E8 in the progression of HCC.

Our in vivo study showed that MFG-E8 overexpression significantly increased the tumor volume/weight and the number of proliferating cells as well as endothelial cells in the tumor grafts. Although the size and histochemical differences of tumors were significantly different between control and MFG-E8-overexpressing groups, the small number of animals is a major limitation in the present study. Further studies involving large numbers of animals in different stages of HCC are needed to elucidate the expression kinetics and the exact mechanism of MFG-E8 in HCC progression.

## 4. Methods and Materials

### 4.1. Tissue Preparation and Cell Culture

Thirty-three pairs of HCC and adjacent non-tumor liver tissues were obtained from HCC patients (n = 33) who underwent surgical resection without any radiotherapy and chemotherapy before the surgery at Kyung Hee University Hospital at Gangdong (Seoul, Korea; IRB No., KHNMC IRB 2012-065). The total of 33 HCC samples included 20 primary and 13 metastatic specimens. The diagnosis of HCC and pathological grades were confirmed by certified pathologists. Pathologic differentiation of primary HCC was classified as grade I-IV according to the Edmondson–Steiner’s histopathological classification [31] and the grades were divided into two groups, the low (grade I and II, n = 10) and high grade (grade III and IV, n = 10) groups. Based on the WHO classification, 1, 17, and 2 of the 20 primary tumor samples were graded as well-, moderately-, and poorly-differentiated tumors, respectively. Human HCC cell lines (Huh7, HepG2, and Hep3B) were obtained from the American Type Culture Collection (USA). All cell lines were cultured in Dulbecco’s modified Eagle’s medium (Hyclone, USA) containing 10% fetal bovine serum (Gibco-BRL, USA) and 1% penicillin/streptomycin (Gibco-BRL) at 37°C in a humidified atmosphere with 5% CO_2_. Human primary hepatocytes were purchased from Lonza (USA) and cultured in Lonza hepatocyte culture media according to the manufacturer’s instructions.

### 4.2. Lentiviral Transduction

Lentiviral pcLV-CMV-MFG-E8-EF1a-Neo-eGFP vector (Sirion Biotech GmbH, Germany) was used to express GFP-tagged MFG-E8 (MFG-E8-GFP) in HCC cell lines. Cells were cultured at 5 × 10^4^ cells/well into 6-well tissue culture plates overnight. The viral supernatant was then added to cells at a multiplicity of infection of 10 with 5 μg/mL polybrene. The expression of GFP was evaluated by fluorescence microscopy to estimate infection efficiency. HCC cells infected with an MFG-E8-expressing lentiviral vector and non-targeting control lentivirus vector were named ‘LV-MFG-E8’ and ‘LV-NTC,’ respectively.

### 4.3. MFG-E8 siRNA, Activity-Neutralizing Anti-MFG-E8 Antibody, and GRGDSP Peptides

Knockdown of MFG-E8 was conducted using Silencer Select predesigned MFG-E8 siRNA or a siRNA negative control from Life Technology (siRNA ID: S224038, Ambion, USA). The siRNA target sequences are as follows: sense 5′-GUGGGUAACUGGAACAAAtt-3′ and antisense 5′-UUUUGUUCCAGUUACCCACaa-3’. HCC cells were seeded at 2 × 10^5^ cells/well in 6-well plates 24 h before transfection. siRNA (50 nM/well) was transfected using LipoJet In Vitro Transfection Kit (SignaGen Laboratories, Rockville, MD, USA). To evaluate whether the neutralization of MFG-E8 activity affected the biological processes of HCC cells, an MFG-E8 activity-neutralizing antibody (sc8029, Santa Cruz, 1 µg/mL) was used for the cellular proliferation and wound healing assays. To evaluate the MFG-E8-mediated integrin signaling, a synthetic integrin-blocking RGD-based peptide (GRGDSP, AnaSpec, Fremont, CA, USA, 100 μM) was used to inhibit integrin-biding to RGD motif of MFG-E8.

### 4.4. Immunostaining

For colorimetric staining, tissue samples were fixed in formalin, embedded in paraffin, and cut into 4-μm-thick sections. Tissue sections were deparaffinized twice in xylene and microwaved for antigen retrieval in low-pH buffer (pH 6.0) for 20 min. After treatment with 3% hydrogen peroxide for 15 min to block endogenous peroxidases, the sections were incubated with the corresponding primary antibodies at room temperature for 15 min, followed by incubation with HRP-labeled polymer-conjugated goat anti-mouse IgG (Dako Cytomation Envision^+^ System, DAKO, Carpinteria, CA, USA) for 30 min. Subsequently, the sections were developed using 3,3’-diaminobenzidine chromogen and substrate buffer (DAKO). All sections were counterstained with hematoxylin. Primary antibodies were purchased from Santa Cruz Biotechnology ( Santa Cruz, CA, USA) and used at the following dilutions: anti-MFG-E8 (sc8029, 1:100), Ki67 (sc23900, 1:100), and AFP (sc8108, 1:50).

For immunofluorescence staining, cells and tissues were fixed in cold 4% paraformaldehyde (Sigma-Aldrich, St Louis, MO, USA) in phosphate-buffered saline (PBS, Gibco-BRL, Gaithersburg, MD, USA). Tissues were embedded in O.C.T. compound (Tissue Tek, Sakura Finetek USA, Torrance, CA, USA) after fixation and then sectioned into 4-μm slices. After blocking and permeabilization in 0.3% Triton X-100 (Sigma-Aldrich) and 10% donkey serum (Sigma-Aldrich) in 0.1% bovine serum albumin (Sigma-Aldrich)/PBS, the sections were incubated at 4 °C overnight with primary antibodies. For MFG-E8, AFP, and CD31 staining, Alexa Fluor 488-conjugated donkey antibodies against rabbit or goat IgG, or Alexa Fluor 594-conjugated donkey antibody against mouse IgG (Invitrogen, Carlsbad, CA, USA) were used as secondary antibodies. An Apotome-Axiovert 200M fluorescence microscope (Carl Zeiss, Oberkochen, Germany) was used to visualize cells after counterstaining with 4′,6-diamidino-2-phenylindoledihydrochlorid (DAPI, 1:1,000, Sigma-Aldrich). To quantify the immunofluorescence intensity of MFG-E8 and AFP expression, ImageJ software (National Institutes of Health, Bethesda, MD, USA) was used to assess the mean fluorescence intensity of greyscale images in five random fields of tumor and normal regions from each tissue section obtained from the 33 patients. The value was expressed as relative values to those in normal tissues, which were arbitrarily set as 1.

### 4.5. ELISA

Culture media were collected from primary hepatocytes and from Huh7, HepG2, and Hep3B cells after 24 h of culture, centrifuged to obtain the supernatants, and assessed for MFG-E8 levels using ELISA kits (R&D Systems, Minneapolis, MN, USA) according to the manufacturer’s instructions. Absorbance was immediately read at 450 nm using a microplate reader (Bio-Tek Instruments, Bad Friedrichshall, Germany).

### 4.6. RT-qPCR

For gene expression analyses, HCC and adjacent non-tumor liver tissues from patients (n = 10) were provided by the Biobank of Ajou University Hospital and Korea University Guro Hospital. Total RNA was extracted from two groups of tissues (HCC and non-tumor tissues) frozen in liquid nitrogen or cultured cells using Trizol reagent (Invitrogen, USA) and then reverse-transcribed into cDNA using the RevertAid First Strand cDNA Synthesis Kit (Thermo Fisher Scientific, Waltham, MA, USA). For RT-qPCR, cDNA was mixed with the appropriate primers and the SYBR-Green Super mix (Bio-Rad, Hercules, CA, USA) and then run on the CFX96 Real-Time System (Bio-Rad). The reaction conditions were 96 °C for 1 min, and 40 cycles of 96 °C for 10 s, 57 °C for 10 s, and 72 °C for 30 s. The 2^–∆∆Ct^ method was used to calculate the relative expression of MGF-E8, and the data obtained were normalized to GAPDH levels. The primer pairs used were: MFG-E8 forward primer, 5′-CCGTAACTTTGGCTCTGTCC-3′ and reverse primer, 5′-TCTTGTGGGAGTGGTTGTCC-3′; Cyclin D1 forward primer, 5′-GCTGCGAAGTGGAAACCATC-3′ and reverse primer, 5′-CCTCCTTCTGCACACATTTGAA-3′; GAPDH forward primer, 5′-AGGGCTGCTTTTAACTCTGGT-3′ and reverse primer, 5′-CCCCACTTGATTTTGGAGGGA-3′.

### 4.7. Western Blot Analysis

Cells were lysed using RIPA buffer for protein extraction. The protein concentration was measured using the bicinchoninic acid (BCA) protein assay kit (Bio-Rad, USA). An equal amount of protein was separated by 8% SDS-PAGE and then transferred onto PVDF membranes (Millipore, Billerica, MA, USA). The membranes were blocked with Tris Buffered Saline-0.1% Tween 20 (TBST)-3% bovine serum albumin (Sigma-Aldrich) and then incubated overnight at 4 °C with primary antibodies against p-AKT (rabbit polyclonal antibody, 1:1000, Cell Signaling Technology, Inc., Danvers, MA, USA), AKT (rabbit polyclonal antibody, 1:1000, Cell Signaling Technology, Inc.), and glyceraldehyde 3-phosphate dehydrogenase (GAPDH; rabbit polyclonal antibody, 1:2000, Santa Cruz Biotechnology). Subsequently, the membranes were washed with TBST solution and then incubated with HRP-conjugated secondary antibody (1:2000). Protein bands were visualized with Pierce™ Fast Western Kit (Thermo Fisher Scientific) and quantified by ImageJ software (National Institutes of Health).

### 4.8. Cell Proliferation Assay

Cells were seeded in each well of a 96-well plate at a density of 1 × 10^4^ cells/well. Following incubation at 37 °C for different periods of time (0, 24, 48, and 72 h), the culture media were removed, and 3-(4,5-dimethylthiazol-2-yl)-2,5-diphenyltetrazolium bromide (MTT) (Sigma-Aldrich, 5 mg/mL) was added to each well. After incubation at 37 °C for another 4  h, the MTT solution was removed and replaced with dimethyl sulfoxide (Sigma-Aldrich). Absorbance was measured at 570 nm using a microplate reader (Bio-Tek Instruments). 

### 4.9. Wound Healing Assay

To determine the role of MFG-E8 in the regulation of HCC cell migration, a wound scrape assay was performed. Briefly, artificial vertical scratches were made on confluent monolayers in 60-mm culture dishes with a 200-μL yellow pipette tip. The process by which the scratches were filled by migrating cells was monitored under a phase-contrast microscope equipped with a digital camera at 0, 24, 48, 72, and 96 h after plating. The cell migration rate was calculated using the following formula: Cell migration distance/original wound width × 100 for each time point.

### 4.10. Mouse Xenograft Model

The animal protocol was approved by the Animal Care and Use Committee of Korea University (IRB No., KUIACUC-2018-0019). Male BALB/c nude mice (6 weeks of age) were purchased from Orient Bio (Gyonggi-Do, Korea). Huh7 cells transfected with LV-NTC (n = 3) or LV-MFG-E8 (n = 3) (both groups: 5 × 10^6^ cells per mouse) were subcutaneously injected into the flanks of BALB/c nude mice. An additional 10 animals were injected with Hep3B (LV-NTC, n = 3; LV-MFG-E8, n = 3) and HepG2 cells (LV-NTC, n = 2; LV-MFG-E8, n = 2). The lengths and widths of tumors were measured using a caliper every week. All mice were euthanized at 9 weeks post-injection, and the tumor nodules were removed and weighed. The tumor volume was calculated according to the following formula: tumor volume (mm^3^) = length (mm) × width (mm)^2^/2. 

### 4.11. Statistical Analysis

All the results except in vivo data were obtained from at least three independent experiments unless otherwise indicated and expressed as the mean ± standard deviation (S.D.). Statistical analyses were performed using SPSS software, version 25.0 (IBM SPSS, Armonk, NY, USA). Comparisons between different groups were made using a two-tailed Student’s t-test. P values less than 0.05 were considered statistically significant.

## 5. Conclusions

HCC is a malignant tumor with high mortality, poor prognosis, and frequent relapse. The results of the present study, to the best of our knowledge, provide the first direct evidence that MFG-E8 substantially contributes to the progression of HCC. All data obtained from human HCC tissues, HCC cell lines, and a HCC xenograft model support this finding. Although the exact mechanism of action remains to be elucidated, our study suggests that targeting MFG-E8 may serve as a promising therapeutic approach against HCC.

## Supplementary Materials

The following are available online at https://www.mdpi.com/2072-6694/12/2/403/s1, Figure S1: Effects of MFG-E8 overexpression on migration of HepG2 and Hep3B cells, Figure S2: Effects of MFG-E8 activity inhibition on HepG2 cell migration, Figure S3: Effects of MFG-E8 activity inhibition on Hep3B cell migration, Figure S4: Effects of MFG-E8 knockdown on HepG2 and Hep3B cell migrations, Figure S5: Growth of tumors in HCC xenograft animals after injections of MFG-E8 overexpressing Hep3B and HepG2 cells.
