# Supplementary material for: Milk Fat Globule-EGF Factor 8 Contributes to Progression of Hepatocellular Carcinoma
Source: Cancers (Basel). 2020 Feb 10;12(2):403. doi: 10.3390/cancers12020403 (PMC7072366; doi:10.3390/cancers12020403)
Supplement: Supplementary file 1 [file cancers-12-00403-s001.zip › cancers-707771-supplementary materials_Rev.docx]

***Supplementary Materials***

Milk fat globule-EGF factor 8 contributes to progression of hepatocellular carcinoma

Duck Sung Ko ^1^, Su Hyun Kim ^2^, Ji Young Park ^1^, Gyunggyu Lee ^1^, Hyo Jin Kim ^1^, Gyeongmin Kim ^1^, Kyun You Chi ^1^, Ilsoo Kim ^1^, Jinseok Lee ^1^, Kyu-Yeoun Won ^2^, Jiyou Han ^3^, Jeongsang Son ^1^, Dong-Hun Woo ^4^, Choongseong Han ^4^ and Jong-Hoon Kim ^1,^*

^1^ Laboratory of Stem Cells and Tissue Regeneration, Department of Biotechnology, College of Life Sciences and Biotechnology, Korea University, Seoul, 02841, Korea; koducksung@gmail.com (D.S.K.); [prssjy@hanmail.net](mailto:prssjy@hanmail.net) (J.Y.P.); [chloe.gyu@gmail.com](mailto:chloe.gyu@gmail.com) (G.L.); [010hyojin@naver.com](mailto:010hyojin@naver.com) (H.J.K.); [kkkjjj0815@naver.com](mailto:kkkjjj0815@naver.com) (G.K.); [viclavoc@naver.com](mailto:viclavoc@naver.com) (K.Y.C.); [kis8295@gmail.com](mailto:kis8295@gmail.com) (I.K.); [wlstjr0806@naver.com](mailto:wlstjr0806@naver.com) (J.L.); [sllzzz@naver.com](mailto:sllzzz@naver.com) (J.S.)

^2^ Department of Pathology, Kyung Hee University Hospital at Gangdong, College of Medicine, Kyung Hee University, Seoul, 05278, Korea; [wonlover2@hanmail.net](mailto:wonlover2@hanmail.net) (S.H.K.) ; [wonki96@hanmail.net](mailto:wonki96@hanmail.net) (K.Y.W.)

^3^ Department of Biological Sciences, Hyupsung University, Hwasung-si, 18330, Korea; [hanjiyou12@hanmail.net](mailto:hanjiyou12@hanmail.net)

^4^ Laboratory of Stem Cells, NEXEL Co., Ltd., Seoul, 07802, Korea; [dhwoo@nexel.co.kr](mailto:dhwoo@nexel.co.kr) (D.H.W); [nexelceo@nexel.co.kr](mailto:nexelceo@nexel.co.kr) (C.H.)

***** Correspondence: [jhkim@korea.ac.kr](mailto:jhkim@korea.ac.kr); Tel.: +82-2-3290-3007

**1. Supplementary Figures**

**1. 1. Supplementary Figure 1**


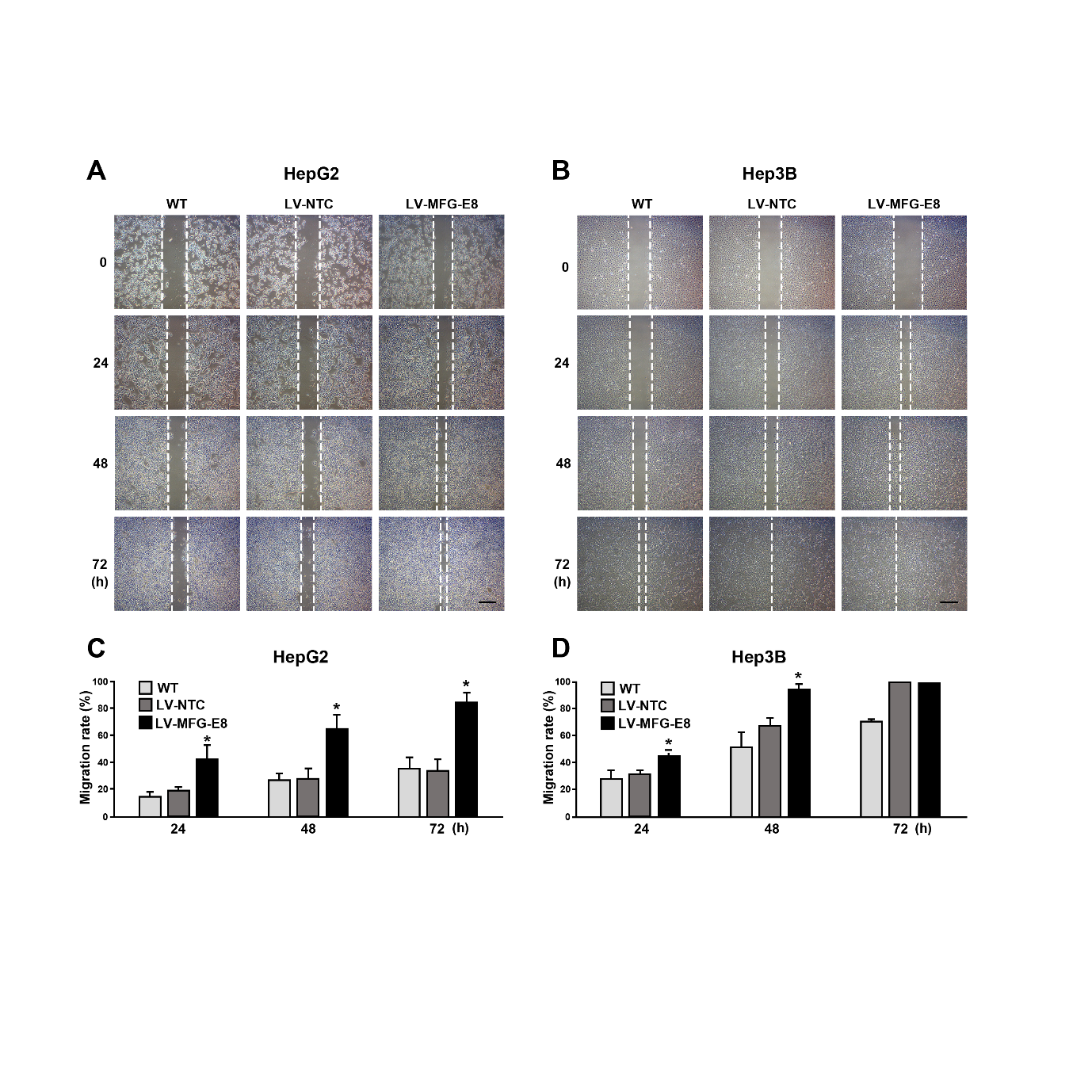


**Figure S1. Effects of MFG-E8 overexpression on migration of HepG2 and Hep3B cells.** Migration of HepG2 (**A**) and Hep3B cells (**B**) transfected with either LV-NTC or LV-MFG-E8 was evaluated by a scratch assay; phase-contrast images were taken 24, 48, and 72 h after making the mechanical scratch wound *in vitro*. The migration rate of HepG2 and Hep3B cells are calculated and separately shown in (**C**) and (**D**), respectively. Data represent the mean ± S.D. **P* < 0.05 by a two-tailed Student’s *t*-test. Scale bars, 200 μm.

**1. 2. Supplementary Figure 2**


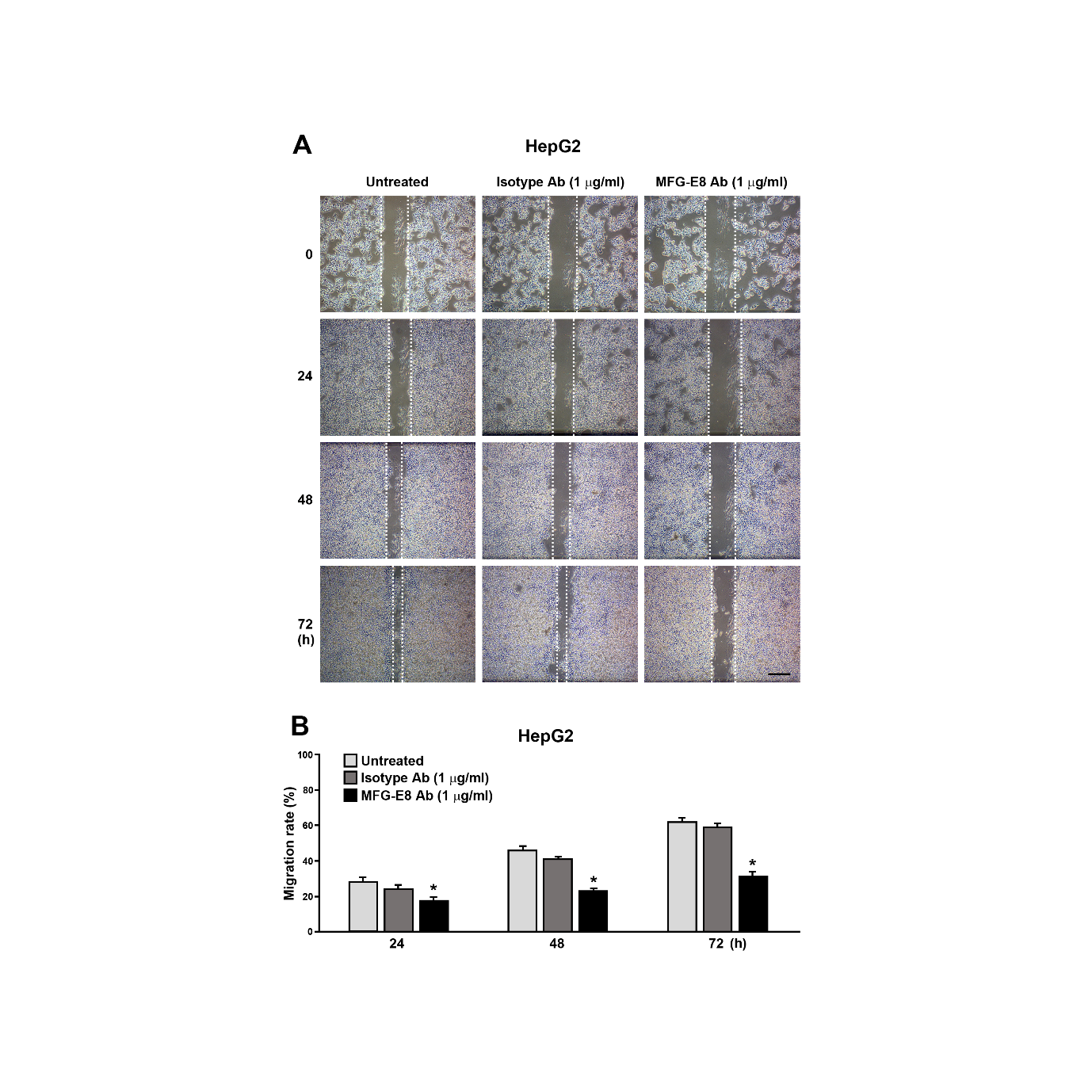


**Figure S2. Effects of MFG-E8 activity inhibition on HepG2 cell migration.** (**A**) Migration of HepG2 cells treated with either isotype IgG or anti-MFG-E8 antibody was evaluated by a scratch assay; phase-contrast images were taken 24, 48, and 72 h after making the mechanical scratch wound *in vitro*. The migration rate of HepG2 cells are calculated and separately shown in (**B**). Data represent the mean ± S.D. **P* < 0.05 by a two-tailed Student’s *t*-test. Scale bars, 200 μm.

**1. 3. Supplementary Figure 3**

**
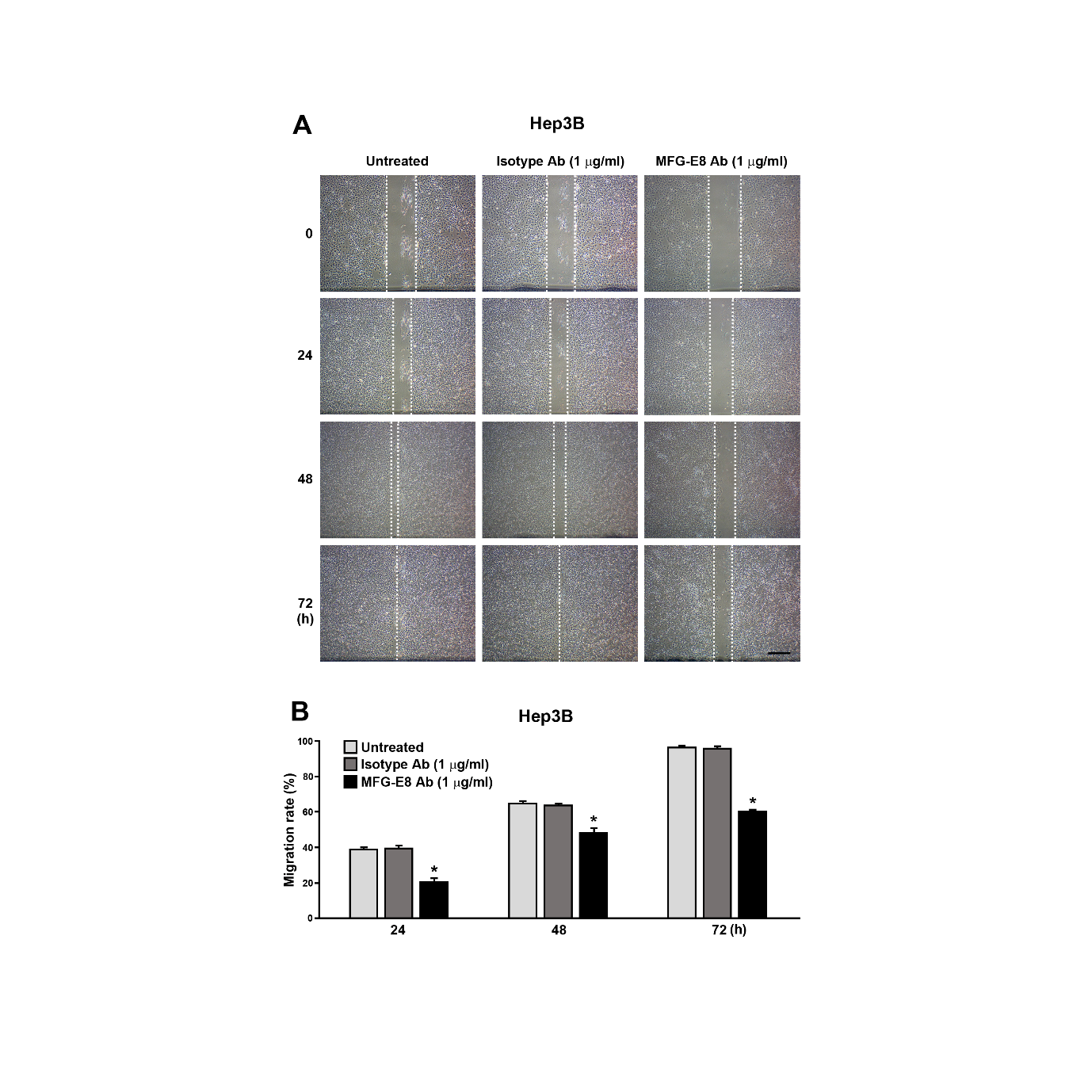
**

**Figure S3. Effects of MFG-E8 activity inhibition on Hep3B cell migration.** (**A**) Migration of Hep3B cells treated with either isotype IgG or anti-MFG-E8 antibody was evaluated by a scratch assay; phase-contrast images were taken 24, 48, and 72 h after making the mechanical scratch wound *in vitro*. The migration rate of Hep3B cells are calculated and separately shown in (**B**). Data represent the mean ± S.D. **P* < 0.05 by a two-tailed Student’s *t*-test. Scale bars, 200 μm.

**1. 4. Supplementary Figure 4**

**

**

**Figure S4. Effects of MFG-E8 knockdown on HepG2 and Hep3B cell migrations.** MFG-E8 expression in HepG2 (**A**) and Hep3B (**B**) cells was silenced by MFG-E8 siRNA, and the rate of migration was compared with that of untransfected (untreated) and nontargeting control siRNA (siRNA-NTC)-transfected HepG2 and Hep3B cells 48 h after making the scratches. Cells transfected with MFG-E8 siRNA were culture in the absence or presence of recombinant human MFG-E8 proteins (rhMFG-E8.) The comparisons of the migration rate are shown on the right. Data represent the mean ± S.D. **P* < 0.05 by a two-tailed Student’s *t*-test. Scale bars, 200 μm.

**1. 5. Supplementary Figure 5**

**
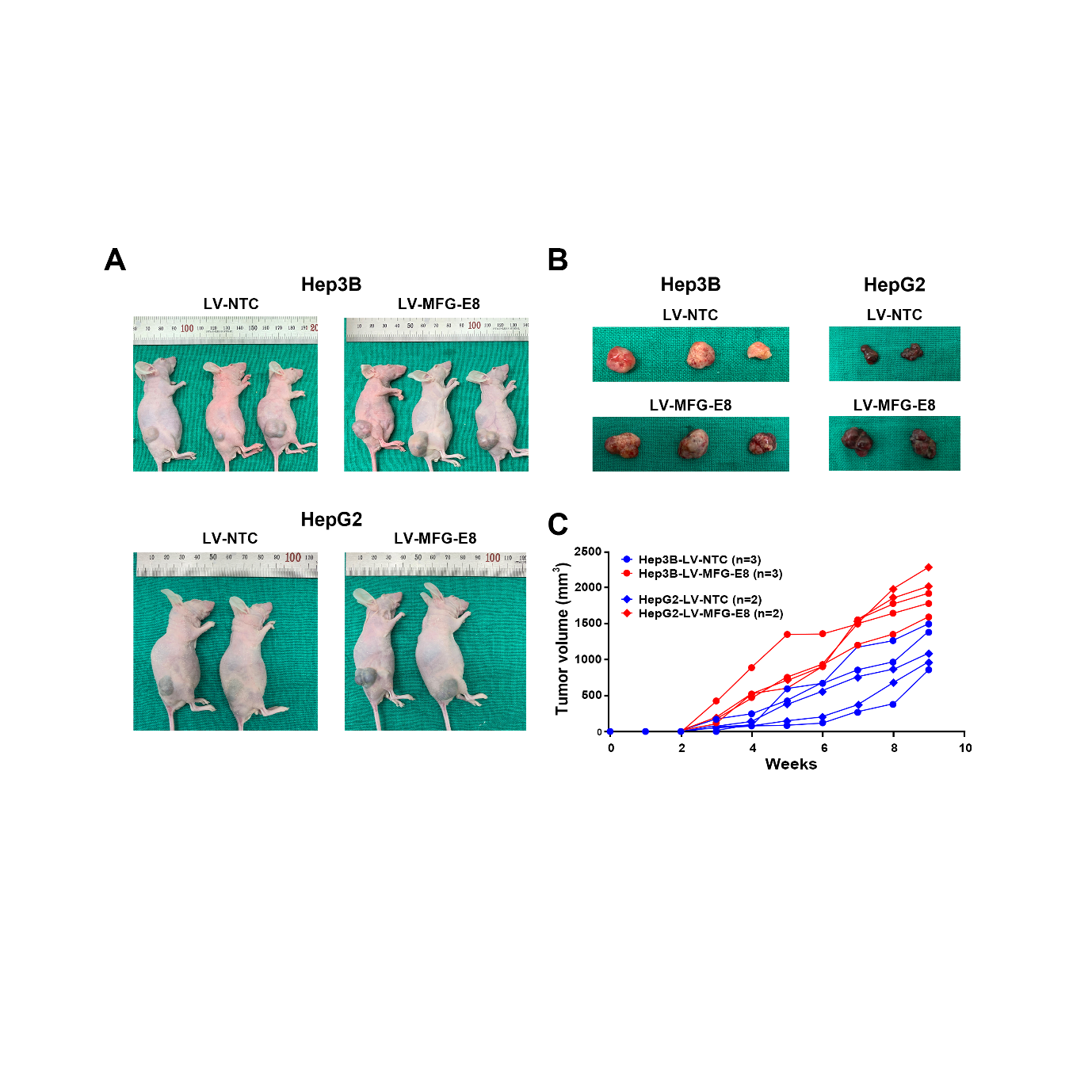
**

**Figure S5. Growth of tumors in HCC xenograft animals after injections of MFG-E8 overexpressing Hep3B and HepG2 cells.** (A) *In vivo* visualization of solid tumors in xenograft mice that received Hep3B cells transfected with LV-NTC or LV-MFG-E8 (upper panels), and mice that received HepG2 cells transfected with LV-NTC or LV-MFG-E8 (lower panels). Images were taken 9 weeks after grafting. (B) Dissected xenografts generated from Hep3B (left panels) and HepG2 cells (right panels) transfected with LV-NTC or LV-MFG-E8. (C) Tumor volume was measured using a caliper every week for 9 weeks.
